# Supplementary material for: Proteomic Changes in Response to Colorless nonripening Mutation during Tomato Fruit Ripening
Source: Plants (Basel). 2022 Dec 17;11(24):3570. doi: 10.3390/plants11243570 (PMC9782875; doi:10.3390/plants11243570)
Supplement: Supplementary file 1 [file plants-11-03570-s001.zip › plants-2038015-supplementary.pdf]

Supplementary Materials

Table S1. The information of all identified proteins acquired by iTRAQ in AC and *Cnr* fruits.

Accession: the accession number of non-redundant protein database of NCBI; Expt. Mr: Experimental molecular mass; Coverage: Amino acid sequence coverage for the identified proteins; Sequence: The sequence information of identified peptides; Score: Mascot scores are statistically significant (p<0.05); COG: Clusters of Orthologous Groups of proteins; KEGG: The Kyoto Encyclopedia of Genes and Genomes; EC: Enzyme Commission; GO: Gene Ontology.

Table S2. The information of DEPs between AC and *Cnr* fruits at the breaker stage.

Accession: the accession number of non-redundant protein database of NCBI; Expt. Mr: Experimental molecular mass; Coverage: Amino acid sequence coverage for the identified proteins; Sequence: The sequence information of identified peptides; Score: Mascot scores are statistically significant (p<0.05); COG: Clusters of Orthologous Groups of proteins; KEGG: The Kyoto Encyclopedia of Genes and Genomes; EC: Enzyme Commission; GO: Gene Ontology. The green or orange background indicates that the DEPs were down or up-regulated respectively in *Cnr* fruit.

Table S3. The information of DEPs between AC and *Cnr* fruits at 10 DPB stage.

Accession: the accession number of non-redundant protein database of NCBI; Expt. Mr: Experimental molecular mass; Coverage: Amino acid sequence coverage for the identified

proteins; Sequence: The sequence information of identified peptides; Score: Mascot scores are statistically significant ($p<0.05$); COG: Clusters of Orthologous Groups of proteins; KEGG: The Kyoto Encyclopedia of Genes and Genomes; EC: Enzyme Commission; GO: Gene Ontology. The green or orange background indicates that the DEPs were down or up-regulated respectively in *Cnr* fruit.

Table S4. The information of overlap DEPs between AC and *Cnr* fruits at the breaker and 10 DPB stages.

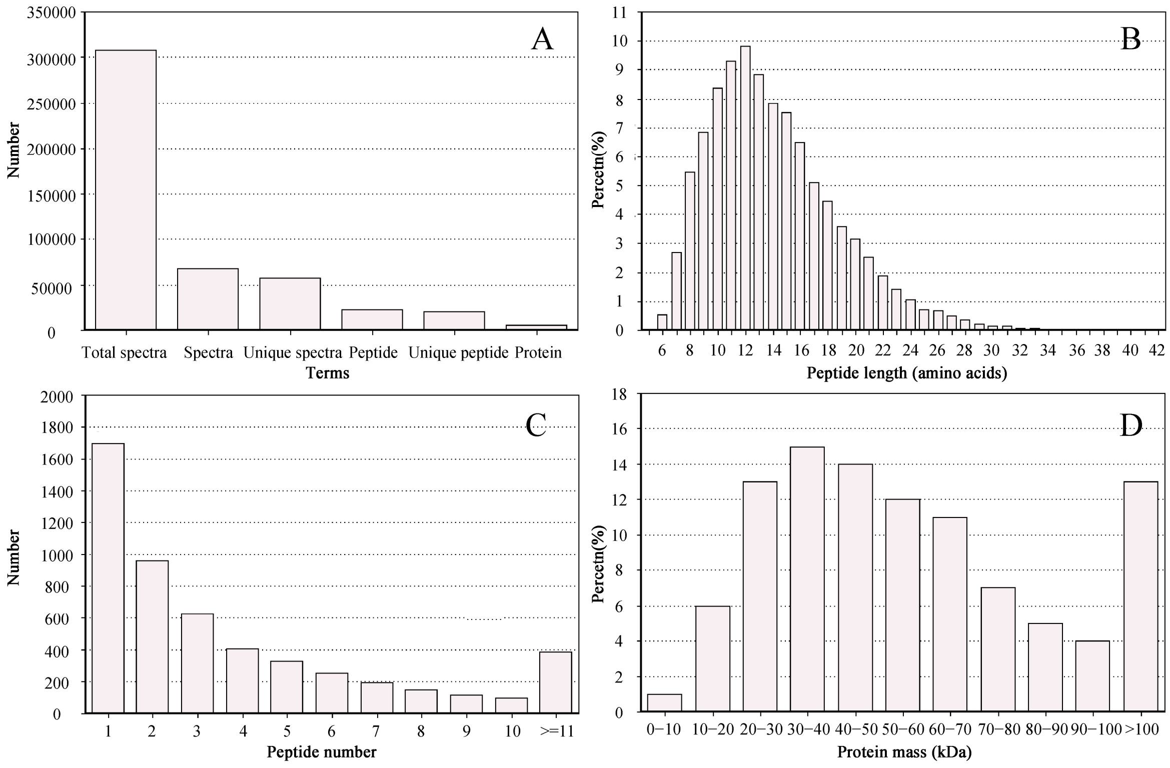

Figure S1 The basic information (A), peptide length distribution (B), peptide number distribution (C), protein mass distribution (D) of total proteins identified by iTRAQ in AC and *Cnr* fruits at different ripening stages.

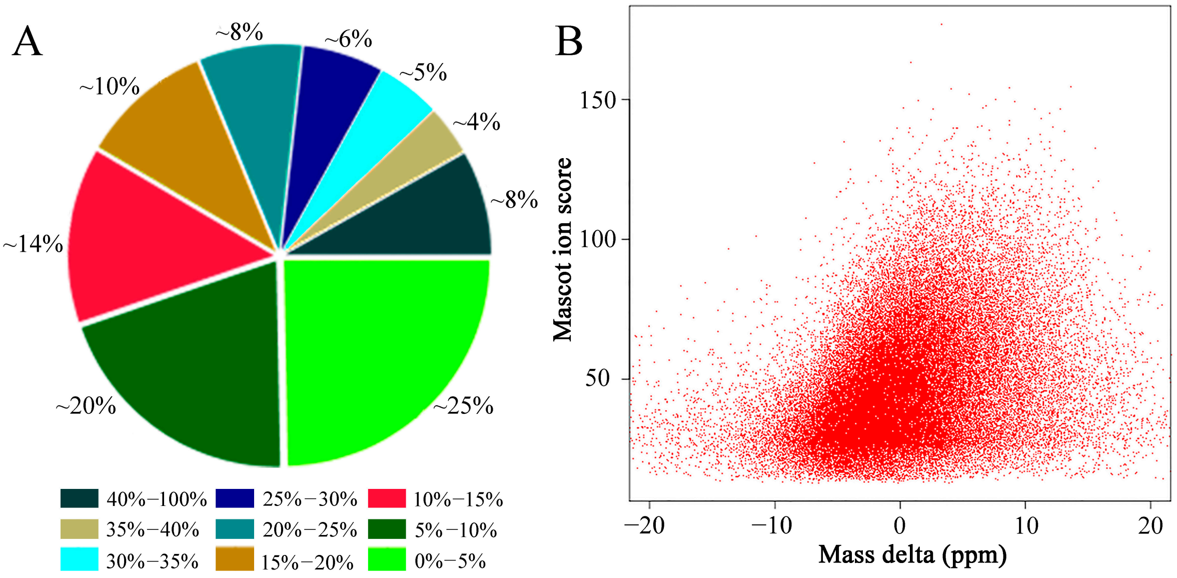

Figure S2 The distribution of protein's sequences coverage (A) and mass error of peptide spectrum matches (B).

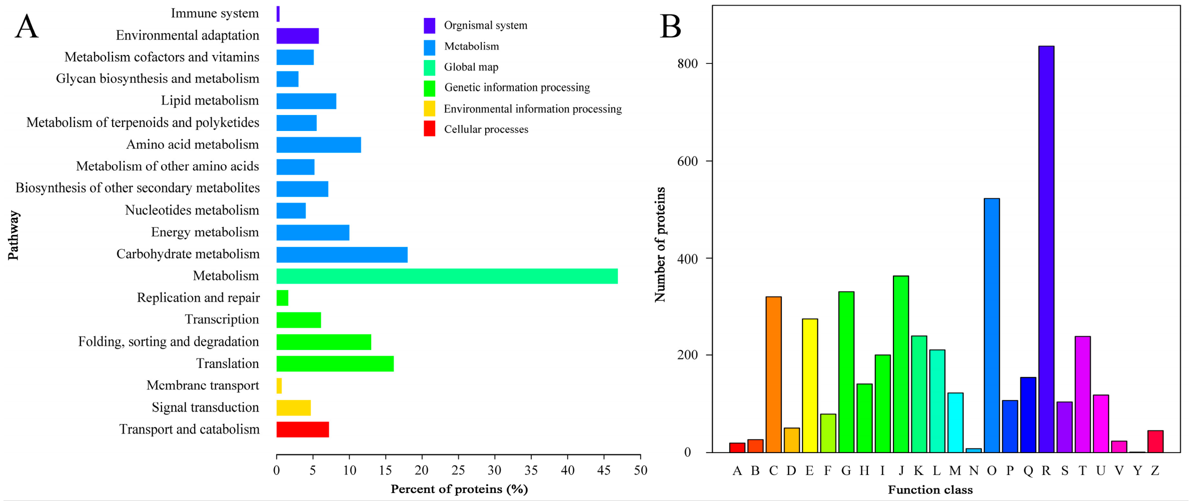

Figure S3 KEGG pathway classification (A) and COG function classification (B) of all identified proteins acquired by iTRAQ. COG function classification are as follows. Function class FC–A: RNA processing and modification; FC–B: chromatin structure and dynamics; FC–C: energy production and conversion; FC–D: cell cycle control, cell division, chromosome partitioning; FC–E: amino acid transport and metabolism; FC–F: nucleotide

transport and metabolism; FC–G: carbohydrate transport and metabolism; FC–H: coenzyme transport and metabolism; FC–I: lipid transport and metabolism; FC–J: translation, ribosomal structure and biogenesis; FC–K: transcription; FC–L: replication, recombination and repair; FC–M: cell wall/membrane/envelope biogenesis; FC–N: cell motility; FC–O: post-translational modification, protein turnover, chaperones; FC–P: inorganic ion transport and metabolism; FC–Q: secondary metabolites biosynthesis, transport and catabolism; FC–R: general function prediction only; FC–S: function unknown; FC–T: signal transduction mechanism; FC–U: intracellular trafficking, secretion, and vesicular transport; FC–V: defense mechanisms; FC–Y: nuclear structure; FC–Z: cytoskeleton.
